# Fabrication of Polyurethane/Polylactide (PU/PLDL) Nanofibers Using Electrospinning Method

**DOI:** 10.3390/ma14092459

**Published:** 2021-05-10

**Authors:** Marta Lech, Joanna Mastalska-Popławska, Jadwiga Laska

**Affiliations:** Faculty of Materials Science and Ceramics, AGH University of Science and Technology, Mickiewicza 30, 30-059 Kraków, Poland; cinnashi@o2.pl (M.L.); jmast@agh.edu.pl (J.M.-P.)

**Keywords:** electrospinning, polymer fibers, polyurethane, polylactide, biomaterials

## Abstract

Polylactide and aliphatic polyurethane are biodegradable synthetic polymers which are broadly used as biomaterials in regenerative medicine for implants and scaffolds for tissue engineering. In this paper, the detailed studies of the fabrication of the electrospun fibers of polyurethane/polylactide mixtures were described. The influence of the used solvent (dimethylformamide (DMF)) and diluents (acetone and dichloromethane (DCM)) on the rheological parameters and electrospinning of the described mixtures was examined. Rheological studies showed that polyure-thane/polylactide mixtures have mostly non-Newtonian character, strongly influenced by the diluent. Solutions containing 50 wt.% or more of polyurethane became less viscous after the addition of DCM or acetone, whereas those with bigger amount of polylactide showed higher viscosity after the addition of DCM and lower viscosity after the addition of acetone. Optimized electrospinning process has been elaborated. Fibers with diameters from 250 nm up to 1 µm have been produced and compared. Pure acetone worsened the electrospinning process, but the more DCM was in the mixture, the thinner and more aligned fibers were produced.

## 1. Introduction

Electrospinning has been patented by Formhals who announced 22 patents on the process in the years 1931–1944 [1]; however, the real academic and industrial interest has arisen only in the beginning of the 21st century [2]. The method gave the opportunity to produce nonwovens and various structures resembling fibroid extracellular matrix or other fibrous tissues [3]. It can be used also in the industry to maintain repeatability and to control the diameter of the produced fibers. This process does not require the use of coagulants or high temperatures to obtain the final product. Other advantages are the economic efficiency of the process and the possibility of producing long length fibers. The disadvantages are mainly the instability of the stream and the produced fibers, what depends not only on the electrospinning parameters, but also on the composition of the polymer solution [4,5].

Forming of fibers by electrospinning occurs in several steps controlled by various physical phenomena. When the cotton fluid flows through the capillary, it becomes charged and initially oriented. The degree of orientation of a stream leaving the capillary is proportional to the capillary length and the flow velocity. This phenomenon is analogous to the observed in the classical process of spinning [6]. Under the high voltage, the droplet becomes stretched until the point when eruption occurs. The stretching is proportional to the voltage [7]. When the critical threshold is reached, a jet is formed and thanks to the cohesion and entanglement of polymer molecules, it maintains its continuity. If the cohesion is not sufficient, electrospraying is observed [8]. The stream of the solution flows for a certain time linearly and the length of this section depends on the electric field forces, the nature of a cotton liquid, the distance to the collector, and a type of a collector [9]. The accumulation of electrostatic charge on the surface of the spun solution causes its rapid acceleration, loss of cohesion, and spiral flow of the stream [10]. Specific selection of the parameters of the electrospinning process can result, for example, in jet branching and, as a consequence, spiky fibers. The final stage of electrospinning is the deposition of the solidified fiber on the collector. In addition, at this stage problems can occur like sticking, cross-linking, deformation of the fibers if the amount of the solvent is too big, or the glass temperature of the polymer is too low, etc. [7].

In our recent research, we studied electrospun polyurethane/polylactide (PU/PLDL) nonwovens for their applicability for nerve regeneration by culturing rat olfactory ensheathing glial cells on them. They were positively assessed [11]. Similar results can be achieved by using a mixture of PU and polycaprolactone (PCL). Separately, these polymers are poorly suited for electrospinning, but in combination they give fibers with very good mechanical properties, which may constitute synthetic vascular scaffolds [12]. To obtain durable materials, PCL nanofibers can also be carbon coated by dipping etched PCL nanofibers in an aqueous solution of carbon nanotubes (CNTs) [13]. Poly(glycolic acid) (PGA), poly(lactide-co-caprolactone) (PLCL), or natural polymers such as collagen and gelatin or their blends can also be used for nonwoven tissue engineering scaffolds [14]. In this paper, we presented a detailed study on the influence of the solvents, viscosity and other properties of cotton liquids of PU/PLDL on the electrospinning process and the quality of the fibers. The obtaining of nonwovens with specific properties (elasticity, rigidness, etc.) is, in practice, quite difficult, mainly because the polymer stream in the electric field is hardly predictable. Hence, individual studies of cotton liquids are required to determine the optimal conditions of the process, to control and to obtain the intended final effect, e.g., fiber diameter, well-ordered aligned fibers, etc. In this paper, the fabrication of nonwovens of PU/PLDL by electrospinning is described. The goal of the research was to elaborate the proper conditions of electrospinning and repetitive fabrication of uniform aligned fibers, potentially facilitating directional regeneration of nervous tissue. The smallest neurons have the diameter approx. 5 μm, the largest approx. 120 μm. Human axons have the diameters from 0.2 to 20 μm (in invertebrates even to 1 mm) and length from several micrometers to several meters. Nonetheless, the artificial guidance for growing nerve fiber should be as thin as possible [15,16,17,18]. Our aim was to fabricate aligned fibers with the diameter from 200 nm to 1 μm. Appropriate proportions and concentrations of polymers in the cotton liquid, the influence of the applied solvent and diluents were determined.

## 2. Materials and Methods

Poly(L/D,L-lactide) (PLDL), with a molar ratio of L-lactide to DL-lactide 80:20 (PURASORB^®^ PLDL8038) was purchased from PURACbiochem BV, Gorinchem, The Netherlands. Polyurethane for biomedical applications (PU) was purchased from BAYER Material Science Company, Leverkusen, Germany. Both polymers were biocompatible and biodegradable and have been used in the form of granulates with 1–2 mm of (PLDL) or 3–4 mm (PU) granules. N,N dimethylformamide (DMF) applied as a solvent, and dichloromethane (DCM) and acetone applied as diluents, have been purchased from Avantor Performance Materials Poland S.A. Gliwice, Poland. Their properties important for the electrospinning process are listed in Table 1.

### 2.1. Preparation of Cotton Liquids

Polylactide and polyurethane were dissolved separately in DMF by stirring at 45–50 °C for 24 h. in case of PLDL and up to 72 h. in case of PU. The concentration of the polymers in the solutions was in the range of 7% to 15% (*w*/*w*). Then, the solutions were mixed in adequate proportions to obtain different PU/PLDL *w*/*w* ratios: 80/20, 50/50, 20/80. The initial solutions of PU and PLDL were also investigated. Additionally, the DMF solutions were diluted with dichloromethane (DCM) or acetone in different proportions with DMF. Different cotton fluids were prepared for every polymer ratio as well as for PU and PLDL, considering the amount of diluent. The final ratios of diluents to the solvent, and the concentrations of polymers in the obtained cotton liquids are presented in Table 2. The solvent/diluent *v*/*v* ratio in the case of 20/80 mixture and sole PLDL were adjusted to obtain satisfactory fibers (Table 2).

### 2.2. Characterization of Cotton Liquids

Rheological examinations (oscillatory tests) were conducted with the use of an Anton Paar Physica MCR301 rheometer (Anton Paar, Graz, Austria) equipped with a PP50 parallel plate. The gap between the disks was 0.175 mm. The polymer solutions were stored for 24 h before the first rheological testing and they were homogenized before each measurement by stirring with a glass rod.

### 2.3. Electrospinning

Electrospinning of the fibers was carried out in a single needle system at 12 kV voltage in a moisture-controlled air atmosphere. An amount of 1–2 mL of a cotton liquid was placed in a vertical plastic syringe and let to flow gravitationally. Four different needles with the inner diameter of 0.45, 0.6, 0.8, and 1.2 mm were tested for each cotton liquid. All cotton fluids had room temperature or they were heated up to 50 °C immediately before use to obtain a homogenous solution. The distance from the tip of the needle to the collector was 2.5 cm. The removable cylindrical grounded metal collector, covered with aluminum foil, could be rotated or stationary.

## 3. Results and Discussion

### 3.1. Rheology of Cotton Liquids

**Figure 1:**
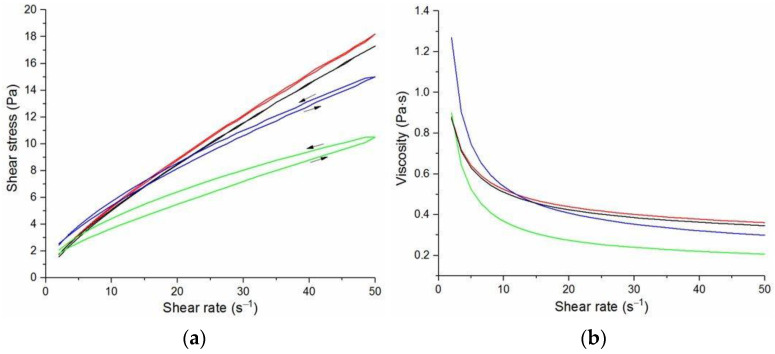
Flow curves (**a**) and viscosity curves; (**b**) of solutions of PU: 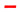
PU/DMF, 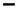
2DMF/1Acetone, 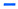
2DMF/1DCM, 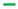
1DMF/1DCM.

**Figure 2:**
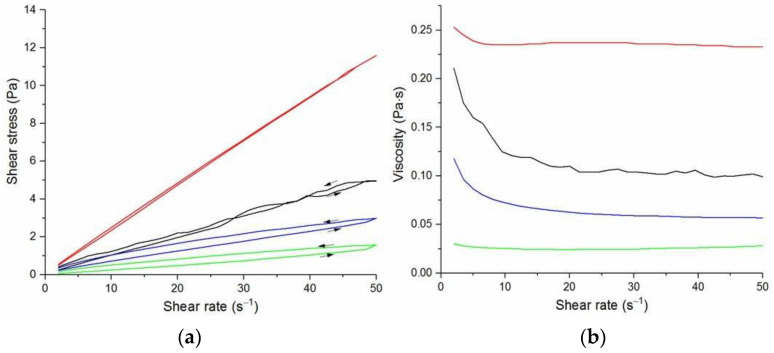
Flow curves (**a**) and viscosity curves; (**b**) of solutions of 80/20 PU/PLDL: 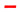
80/20/DMF, 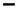
2DMF/1Acetone, 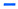
1DMF/1Acetone, 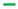
1DMF/2Acetone.

**Figure 3:**
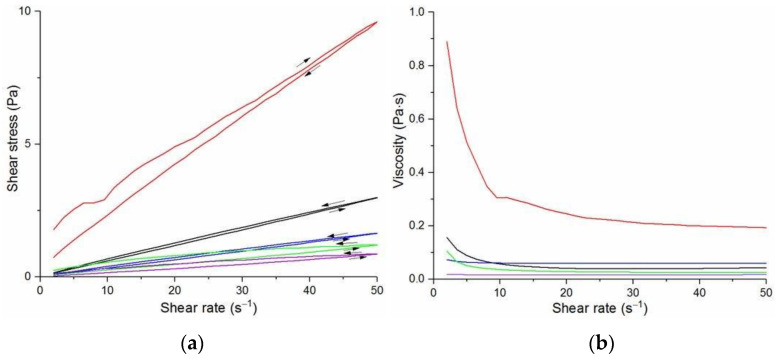
Flow curves (**a**) and viscosity curves; (**b**) of solutions of 50/50 PU/PLDL: 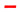
50/50/DMF, 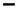
2DMF/1DCM, 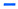
2DMF/1Acetone, 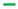
1DMF/1Acetone, 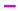
1DMF/2DCM.

**Figure 4:**
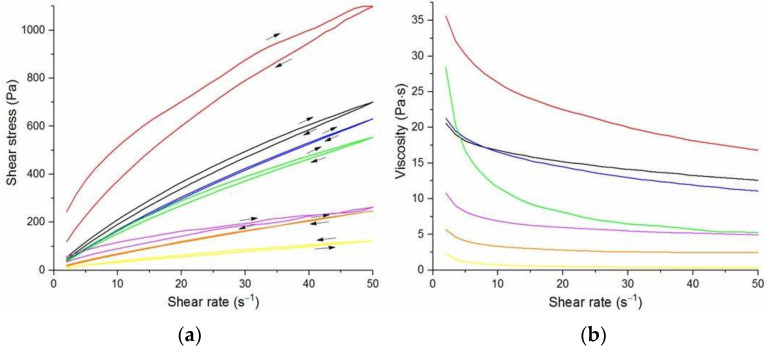
Flow curves (**a**) and viscosity curves; (**b**) of solutions of 20/80 PU/PLDL: 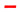
2DMF/1DCM, 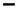
1.4DMF/1DCM, 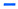
20/80/DMF, 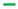
1.1DMF/DCM, 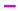
1.44DMF/1Acetone, 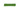
2DMF/1Acetone, 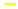
1.1DMF/1Acetone.

**Figure 5:**
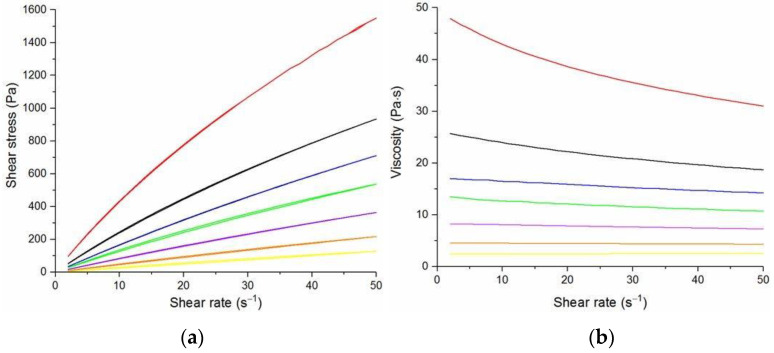
Flow curves (**a**) and viscosity curves; (**b**) of solutions of PLDL: 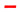
3DMF/1DCM, 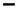
2DMF/1DCM, 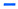
PLDL/DMF, 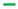
1.5DMF/1DCM, 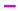
3DMF/1Acetone, 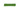
2DMF/1Acetone, 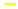
1.5DMF/1Acetone.

The flow curves and viscosity curves of the cotton liquids are shown in Figure 1, Figure 2, Figure 3, Figure 4 and Figure 5. Most cotton liquids prepared for this study showed non-Newtonian characteristics with a flow limit. The flow limit of all solutions increased during storage. The PU, 80/20 and 50/50 solutions showed less or more visible shear thinning (Figure 1, Figure 2 and Figure 3). The flow curves of the DMF solutions of PU and 80/20 showed no hysteresis, while solutions diluted with DCM and acetone were thixotropic. Rheological behavior of 50/50 and 20/80 depended on the content of a solution. On the other hand, PLDL in DMF formed well-defined Newtonian or Bingham solutions both with acetone and DCM as diluents (see Figure 5).

In general, DMF solutions containing 50 wt.%. or more of polyurethane became less viscous after the addition of DCM or acetone. Addition of DCM to PLDL or 20/80 DMF increased their viscosity while acetone caused a decrease in viscosity. It means a strong interaction between DCM with PLDL. The maximum shear stress for PU, 80/20 and 50/50 solutions is less than 20 Pa, while in the case of solutions rich in PLDL (20/80, 0/100) the shear stress reaches up to 1600 Pa. A similar dependence is observed in the case of viscosity. The maximum viscosity for PU, 80/20 and 50/50 solutions is less than 2 Pa·s, while for the rest solutions it reaches almost 50 Pa·s. When hysteresis was observed in the flow curves, it showed thixotropic (100/0, 80/20, 50/50, and PLDL) or antithixotropic (20/80) character of the fluids independently on the content of a diluent. Most of the hysteresis loops were open, indicating the slowness of the process of internal structure stabilization. The solutions containing dichloromethane and these containing no diluents, showed a tendency to gelation forming homogenous thixotropic gels. The gelation was reversible, the reoccurrence of the initial viscosity could be brought back by mechanical stirring or shearing with the rheometer. During storage, the viscosity of the solutions comprising acetone increased faster to those with DCM, probably due to the stronger dipole–dipole interaction between the polymers and the solvent. The gelation was not homogenous, especially in PU reach samples, but occurred with the creation of local compactions (lumps). The creation of lumps caused the need for applying a higher shear stress at a given shear rate compared to the fresh solutions. After 22 days, the lumps became so compact and springy that they were pushed out the oscillating plates during the rheological measurements. The remaining solution between plates had visibly lower viscosity. These changes were also reversible upon heating to 60 °C, even after 4 months. After 3–4 weeks, the character of the flow curves changed, in most cases, to viscoelastic (nonlinear). Due to many data and their complexity, the detailed study of the rheological behavior of the PU/PLDL solutions need a separate description and analysis, and will be presented in another paper.

The solutions of PLDL remained homogenous during 4 weeks of observation. Their flow curves are characteristic for Bingham liquids, do not show any irregularities indicating flocculation, and the viscoplastic region is not present. Solutions of PLDL/DMF diluted with DCM showed clear Bingham character. Their flow curves did not change markedly after aging the solutions. In case of the solutions diluted with acetone, a film on the walls of the flask was created after several days of storage causing a decrease in viscosity of the remaining solution.

### 3.2. Electrospinning of Fibers

**Figure 6:**
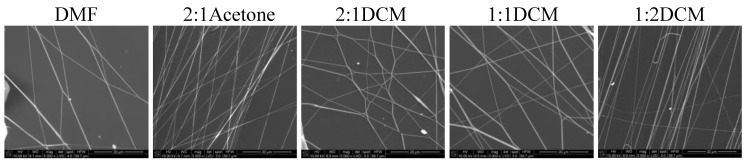
SEM images of electrospun fibers of polyurethane. Needle diameter: DMF, 2:1Acetone, 2:1DCM, 1:1DCM—0.6 mm; 1:2DCM—0.45 mm.

**Figure 7:**
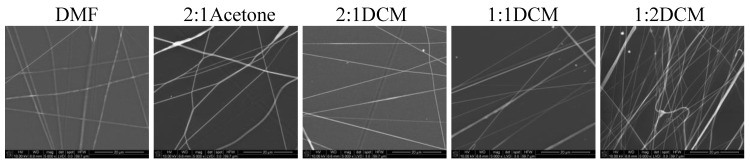
SEM images of electrospun fibers of PU/PLDL 80/20. Needle diameter: DMF—0.8 mm; 2:1Acetone, 2:1DCM—0.45 mm, 1:1DCM, 1:2DCM—0.6 mm.

In Figure 6 and Figure 7 SEM images of nonwovens electrospun from polyurethane rich cotton liquids (100/0 and 80/20) are presented.

The fibers are smooth and devoid of defects. Electrospinning of fibers 100/0 was problematic because the solutions did not create a uniform stream but dripped from the tip of a needle. The solutions diluted with acetone (1DMF/1Acetone and 1DMF/2Acetone) did not bring satisfying results. On one hand, the viscosity of these solutions was too low, on the other, reducing the diameter of the needle to 0.45 mm did not help at all as it got clogged. The lessening of the acetone amount in the cotton liquid to the ratio of 2DMF/1Acetone gave fairly positive results, i.e., irregular and defected fibers have been produced. Use of DCM as a diluent allowed to obtain fibers from the solutions of all DMF/DCM ratios. The more DCM was in the cotton liquid, the thinner and more aligned fibers were produced. The solution PU/1DMF/2DCM gave the thinnest fibers of all in this study, with the average diameter 203.7 ± 71.4 nm. The fibers created from the solutions diluted with DCM tended to aggregation into bundles, while the electrospinning process was fast and trouble-free. As we can see in Table 1, DMF and DCM have higher surface tension compared to acetone, and the surface tension and viscoelasticity of the solutions are the key parameters of electrospinning [19]. Together with a polymer concentration, they determine the fiber diameter and morphology, what is clearly visible in our study. Usually, low surface tension solvents are chosen for electrospinning as the high surface tension requires applying the considerably higher electric potential. Yet, in this case, the high volatility of DCM quickened the solidification of fibers, positively influencing the quality of the fibers. Electrospinning the fibers from the solution of polyurethane in DMF required careful balancing of the process parameters. Due to its relatively high viscosity, the use of thin needles was limited, however, when a larger diameter needle was used the solvent did not vaporize effectively enough. The best quality fibers in the series 80/20/DMF/Acetone were fabricated using the solution 2DMF/1Acetone. 80/20/DMF solutions gave thin fibers but the diameters varied significantly. In turn, fibers from the DMF solution with no diluent had smaller but more varied diameters. Addition of DCM allowed to produce the thinnest fibers. The more DCM in the cotton liquid, the thinner the fibers. The 80/20/1DMF:2DCM solution allowed for fabrication the densest nonwovens. This result is surprising regarding the low dielectric constant of DCM. Usually the cotton liquids based on solvents with high dielectric constant give thin and smooth fibers.

**Figure 8:**
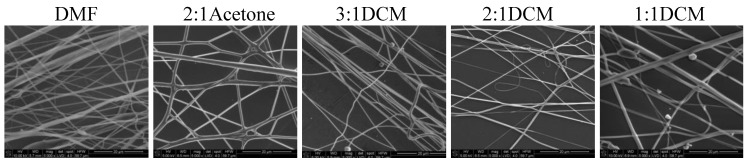
SEM images of electrospun fibers of PU/PLDL 50/50. Needle diameter: DMF, 2:1Aetone, 3:1DCM, 2:1DCM—0.6 mm; 1:1DCM—0.8 mm.

**Figure 9:**
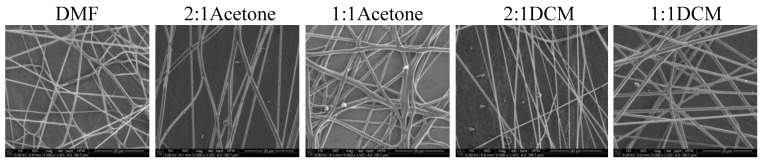
SEM images of electrospun fibers of PU/PLDL 20/80. Needle diameter 0.6 mm.

**Figure 10:**
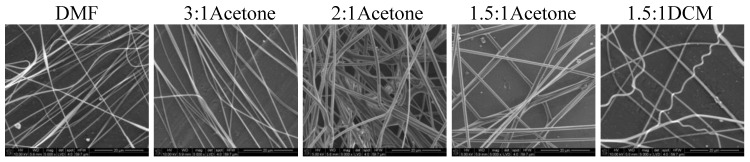
SEM images of electrospun fibers of PLDL. Needle diameter 0.6 mm.

Nonwovens obtained from the solutions of 50/50P, 20/80, and PLDL had considerably larger thickness of fibers than these from PU and 80/20. Tendency to stick together was observed in some cases (Figure 8, Figure 9 and Figure 10). The thickness of the stuck fibers was not taken into consideration. The most quick process was in the case of 50/50 solution which viscosity was the highest of all, causing the necessity of heating the solution up to 40–50 °C before processing. At room temperature, the solution was too viscous to create a continuous stream. As a consequence fibers and small spheres were created. Some deformations of fibers (irregularity, sticking) appeared when the quantity of acetone was larger than 1DMF/1Acetone. When DCM was applied as a diluent, clusters of fibers were created, especially for lower contents of the diluent. The highest number of clusters and tangles was created from 50PU:50PLDL/3DMF/1DCM cotton liquid. Moreover, the surface of the fibers was irregular. However, this solution allowed for creation of the second densest nonwoven after no-diluent cotton liquid. Fibers with the average diameter of 500 nm were created in the case of 50/50/3DMF/1DCM.

The 20PU:80PLDL/DMF solutions were also processed at the temperature of 50 °C. The creation of regularly distributed fibers (nonwovens) with the use of the biggest needle of 1.2 mm in diameter was relatively easy. The addition of acetone caused agglomeration of fibers (see SEM photo of 20/80/1DMF/1Acetone, Figure 9), on the other hand it resulted in rough surface of the fibers, diversified thickness and creation of callosities (beads) on the 20/80/2DMF/1Acetone fibers. The optimum solution in this series, giving a separate non-defected fibers, was 20/80/1.4DMF/1Acetone. Similarly to the other cotton liquids (80/20, 50/50) small amounts of DCM as a diluent gave the best quality nonwovens.

The cotton liquids of polylactide, namely, PLDL/3DMF/1DCM and PLDL/2DMF/1DCM, were too viscous for fiber preparation even in the temp. 50 °C. The solution PLDL/1.5DMF/1DCM produced fibers with irregular surface, and the best results were obtained for PLDL/3DMF/1Acetone. The latter gave smooth, regular fibers, which did not stick together, while higher amounts of acetone caused aggregation of the fibers in bundles.

**Figure 11:**
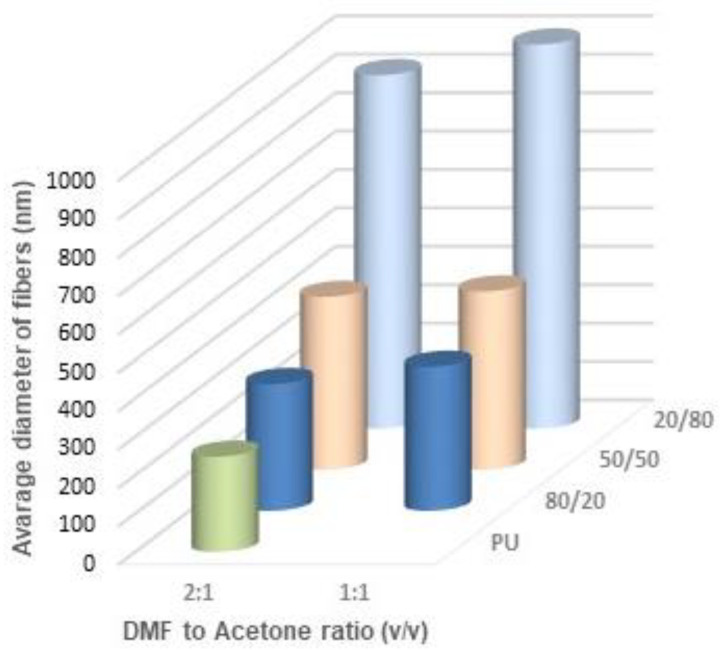
Thickness of the fibers electrospun from cotton liquids diluted with acetone.

**Figure 12:**
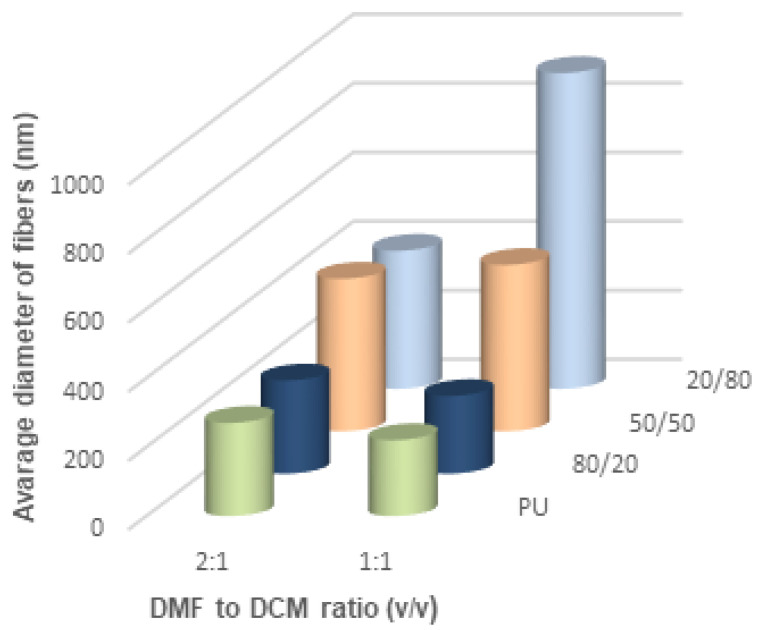
Thickness of the fibers electrospun from cotton liquids diluted with dichloromethane.

**Figure 13:**
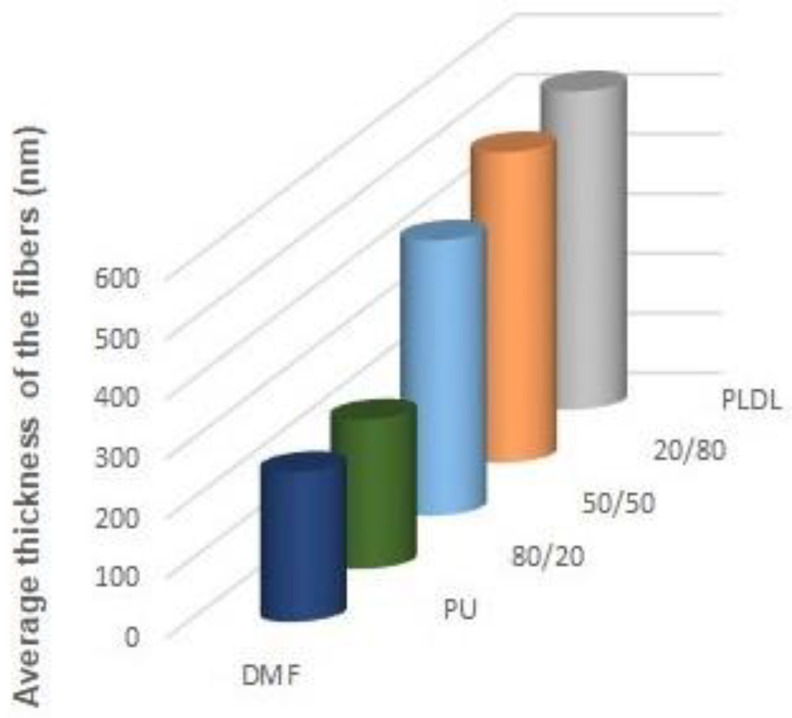
Thickness of the fibers electrospun from DMF cotton liquids without dilution.

Figure 11, Figure 12 and Figure 13 show the average diameters of the fibers dependent on the polymer ratio and solvents. It is clear that the fibers enriched in PLDL are significantly thicker the other ones when acetone was used as a diluent. The DMF acetone ratio did not influence noticeably the thickness of the fibers (Figure 11). When DCM was applied as a diluent, the fiber diameter depended on the DMF/DCM ratio. Equal amounts of solvents caused similar dependence as DMF/Acetone, i.e., fibers of 20/80 PU/PLDL had the diameter ~900 nm while the rest of polymer compositions gave fibers thinner than 400 nm. The cotton liquids of 2DMF/1DCM behaved similarly to DMF solutions. The fibers were thinner than 400 nm.

## 4. Conclusions

Influence of different solvents and diluents (DMF, DCM, and acetone) on the rheological behavior and electrospinning of PU/PLDL cotton liquids was investigated. The flow curves of the DMF solutions of PU, 80/20 and PLDL showed well-defined Newtonian or Bingham character, while the solutions diluted with DCM and acetone show a hysteresis loops, what indicates that the addition of the diluent had a significant impact on the rheological behavior of the cotton liquids. DMF solutions containing 50 wt.% or more of PU became less viscous after the addition of DCM or acetone, whereas those with bigger amount of PLDL (0/100 or 20/80) showed their viscosity increased after the addition of DCM and decreased after the addition of acetone. It means a strong interaction between DCM with PLDL. Most of the PU/PLDL cotton liquids showed non-Newtonian course with a flow limit that increased during storage, despite the used diluent. Viscosity of the stored solutions comprising acetone increased faster to those with DCM, probably due to the stronger dipole–dipole interaction between the polymers and the solvent. Addition of a diluent had a significant impact on the electrospinning process. Pure acetone worsened this process, while DCM allowed to obtain fibers independently on the DMF/DCM ratio. The more DCM was in the cotton liquid, the thinner and more aligned fibers were produced. It is because DMF and DCM have higher surface tension compared to acetone. However, in the case of very viscous solutions, i.e., with a predominance of PLDL, thanks to the addition of acetone, the thickness and the surface of the fibers could be controlled.

## Figures and Tables

**Table 1:** Properties of the solvents and diluents used for electrospinning.

Property	DMF	DCM	Acetone
Boiling Point (°C)	153	40	56
Dynamic Viscosity (mPa·s)	0.8	0.4	0.3
Dielectric Constant	36.7	9.1	20.7
Surface Tension (mJ/m^2^)	36.3	27.8	22.7

**Table 2:** Composition of the investigated cotton liquids.

PU/PLDL *w*/*w* Ratio	Initial Concentration of the Polymers in DMF % (*w*/*w*)	DMF/Diluent Ratio (*v*/*v*)				
		1:0	3:1	2:1	1:1	1:2
100/0	7	+	−	+	+	+
80/20	7	+	−	+	+	+
50/50	7	+	−	+	+	+
50/50	13	+	+	+	+	−
20/80	15	+	−	+	1.4:1	1.1:1
0/100	15	+	+	+	1.5:1	−

## Data Availability

The data presented in this study are available within the article. They are also available on request from the corresponding author.
